# Ciltacabtagene Autoleucel for Patients With Triple-class Exposed Multiple Myeloma: Adjusted Comparison of CARTITUDE-1 Patient Outcomes Versus Real-world Clinical Practice

**DOI:** 10.1097/HS9.0000000000000813

**Published:** 2022-11-29

**Authors:** Michel Delforge, Marie-Christiane Vekemans, Julien Depaus, Nathalie Meuleman, Ann Van de Velde, Isabelle Vande Broek, Sophie Vandervennet, Sandra Van Hoorenbeeck, Evelien Moorkens, Danielle Strens, Joris Diels, Francesca Ghilotti, Benjamin Haefliger, Sander Dalhuisen, William Deraedt, Sébastien Anguille

**Affiliations:** 1 Universitaire Ziekenhuizen Leuven, Belgium; 2 Cliniques Universitaires Saint-Luc, Brussels, Belgium; 3 Department of Haematology, Université catholique de Louvain, CHU UCL Namur, Yvoir, Belgium; 4 Institut Jules Bordet, Université Libre de Bruxelles, Brussels, Belgium; 5 Division of Hematology and Center for Cell Therapy & Regenerative Medicine, Antwerp University Hospital, Edegem, Belgium; 6 Heilig Hartziekenhuis, Lier, Belgium; 7 AZ Nikolaas, Haematology, Sint-Niklaas, Belgium; 8 Janssen-Cilag NV, Beerse, Belgium; 9 Realidad bvba, Grimbergen, Belgium; 10 Janssen Pharmaceutica NV, Beerse, Belgium; 11 Janssen-Cilag SpA, Cologno Monzese, Italy; 12 Cilag GmbH International, Zug, Switzerland; 13 Janssen-Cilag BV, Breda, the Netherlands; 14 Laboratory of Experimental Hematology, Vaccine and Infectious Disease Institute, Faculty of Medicine and Health Sciences, University of Antwerp, Belgium

Multiple myeloma (MM) is a hematological cancer characterized by the clonal proliferation of malignant plasma cells in the bone marrow.^1^ Disease relapse frequently occurs, with treatment commonly involving sequential lines of treatment (LOT) including immunomodulatory agents (IMiD), proteasome inhibitors (PI), and monoclonal antibodies.^2^ While these agents have improved outcomes in patients with MM, this condition remains incurable for the majority of patients.^3^ Patients become refractory to therapy, resulting in a lack of treatment options and a poor prognosis. For triple-class exposed (TCE) MM patients (ie, prior exposure to PIs, IMiDs, and anti-CD38 antibodies), there is no established standard of care and outcomes are poor.^2,4,5^

Ciltacabtagene autoleucel (cilta-cel; JNJ-68284528) is a chimeric antigen receptor T-cell therapy that targets the B-cell maturation antigen.^6^ The safety and efficacy of cilta-cel in TCE MM patients has been evaluated in the single arm, open-label CARTITUDE-1 clinical trial (NCT03548207).^7,8^ In absence of a randomized study, comparisons of outcomes with an external cohort of similar patients can provide vital evidence regarding the benefits of cilta-cel in comparison to treatments currently used in real-world clinical practice (RWCP). BELCOMM (Belgium Comparator study in Multiple Myeloma) represents a retrospective study capturing longitudinal data from March 2017 to May 2021 regarding patient characteristics and outcomes across treatment lines from TCE RRMM patients treated with RWCP from 7 academic and nonacademic centers in Belgium, who fulfilled the main inclusion criteria from CARTITUDE-1 (see Suppl. Section 1). Here we present findings from adjusted comparisons using the RWCP cohort as an external control arm for CARTITUDE-1 to compare response and survival outcomes between cilta-cel and RWCP in patients with TCE RRMM.

Within CARTITUDE-1, 113 patients enrolled from centers in the United States between July 2018 and October 2019 underwent apheresis; the collected T-cells were used to produce cilta-cel. Of these, 97 patients received cilta-cel, while 16 did not receive infusions due to early withdrawal (n = 5), progressive disease (n = 2), or death (n = 9). Analyses were conducted based on the datacut from January 2022 (median follow-up = 27.7 months). The RWCP cohort, included 112 patients (representing 237 LOTs).

Main comparative analyses included the 97 infused patients from CARTITUDE-1 (the *infused population*). As these patients are a subset of the 113 enrolled patients, who were progression free at time of infusion (which occurred on average 52 days after enrollment), in a similar way only patients still progression free at day 52 after treatment initiation (re-defining the index date at start of LOT + 52 days) were included in the comparative analysis, to avoid immortal time bias. This “aligned population” included 90 RWCP patients, with 145 LOTs fulfilling the inclusion criteria of CARTITUDE-1 at treatment initiation (the *aligned population*). A second comparative analysis was performed including all 113 patients enrolled in CARTITUDE-1 (113 LOTs) and the 112 patients who were enrolled in BELCOMM (237 LOTs) (referred to as the *enrolled populations*) (with index dates the day of apheresis and the start of LOT, respectively).

The outcomes compared between groups were overall response rate (ORR), very good partial response rate (≥VGPR), progression-free survival (PFS), time to next treatment (TTNT), and overall survival (OS); for details, see Suppl.Section 1. To adjust for confounding due to imbalances in clinically important prognostic factors, comparative analyses were adjusted for the following baseline characteristics (defined based upon clinical expert input and availability in both cohorts): refractory status, extramedullary disease, time to progression on last regimen, number of prior LOTs, years since MM diagnosis, average duration of prior LOTs, age, sex, lactate dehydrogenase, hemoglobin and albumin (sensitivity analyses additionally included cytogenetic risk, MM type and ECOG). The statistical approach of inverse probability weighting (IPW) was used, which involves 2 steps. First, propensity scores (representing the probability for each of the patients to belong to the CARTITUDE-1 cohort rather than the RWCP cohort, given the baseline factors) were estimated using a logistic regression, and these scores were then transformed into patient-specific weights for the BELCOMM cohort, in such a way that the reweighted BELCOMM cohort was well balanced on all prognostic factors as observed in CARTITUDE-1 (using “ATT” weights, to estimate the “Average Treatment effect in the Treated population” [ie, CARTITUDE-1]). As this reweighted BELCOMM cohort resembles the CARTITUDE-1 population, the comparison versus the observed CARTITUDE-1 cohort can be viewed as mimicking a randomized clinical trial and is expected to be unbiased, so long as no imbalances remain due to unobserved prognostic factors for which adjustments were not possible. Balance between groups was assessed using standardized mean differences (SMD). In a second step, outcomes between cilta-cel and RWCP were compared using weighted logistic regression for binary endpoints (ORR and ≥VGPR) and weighted Cox proportional hazards models for time to event endpoints (PFS, TTNT, and OS); additional details regarding data analysis are provided in Suppl. Section 1. Here we describe first findings within the infused/aligned populations, and then comment on findings from the enrolled populations.

A total of 90 patients (145 LOTs) from the RWCP group were included (see Suppl. Section 2). In 37.9% of the initiated treatment lines, RWCP patients were refractory to 4 or 5 therapies, 57.0% had received ≥5 prior LOTs, 14.5% had extramedullary disease, and in 42.1% of observations the time to progression in the prior LOT was shorter than 4 months. After patients became TCE, >50 unique treatment regimens were used; Suppl. Section 3 summarizes the most common regimens received. Most patients had previously received daratumumab (100.0%), bortezomib (99.3%), and lenalidomide (97.9%), with most patients being refractory to these therapies (totals of 81.4%, 89.0%, and 53.8%). Information regarding medical resource use in the RWCP population is provided in Suppl. Section 4.

IPW-ATT re-weighting significantly reduced imbalances between groups in the infused/aligned populations, with most SMDs <0.20 (see Suppl. Section 5 and Suppl. Section 6).

Of the infused cilta-cel patients, 96.9% reached ORR versus 40.0% and 27.0% in the observed and ATT-weighted RWCP cohorts; corresponding rates for ≥VGPR were 93.8% versus 17.2% and 8.0%, respectively. After IPW-ATT adjustment, patients on cilta-cel were 3-fold and 11.5-fold as likely to reach ORR (RR 3.00 [95% CI, 2.32-3.89]; *P* < 0.0001) and ≥VGPR (RR 11.51 [6.38-20.77]; *P* < 0.0001), respectively. Similar findings were observed for the enrolled population (Figure 1B).

**Figure 1.**
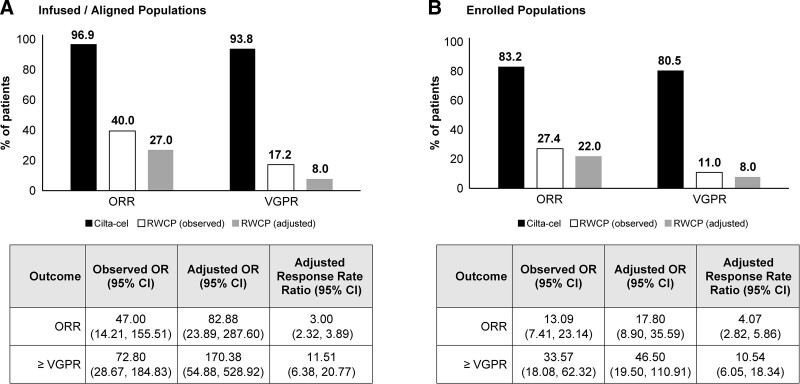
**Summary of observed and adjusted rates of clinical response.** Observed and adjusted data comparing rates of clinical response between cilta-cel and RWCP in the infused/aligned populations and the enrolled populations are presented. Adjusted comparisons account for the effects of refractory status, extramedullary disease, time to progression on last regimen, number of prior LOTs, years since MM diagnosis, average duration of prior LOTs, age, sex, lactate dehydrogenase, hemoglobin and albumin. All comparisons favored cilta-cel. Analyses of infused/aligned populations consisted of 97 lines of therapy for cilta-cel and 145 for RWCP, while analyses of enrolled populations consisted of 113 lines of therapy for cilta-cel and 237 lines of therapy for RWCP. ATT = average treatment effect in the treated population; IPW = inverse probability weighting; OR = odds ratio; ORR = overall response rate; RWCP = real-world clinical practice; VGPR = very good partial response.

Median PFS was not yet reached in the cilta-cel cohort, while the observed and the ATT-reweighted median PFS for the RWCP cohort were 3.9 and 2.9 months, respectively. Figure 2A presents the unadjusted and adjusted Kaplan-Meier curves for PFS for both cohorts; the adjusted hazard ratio (HR) for cilta-cel versus RWCP was 0.12 [0.07-0.18].

**Figure 2.**
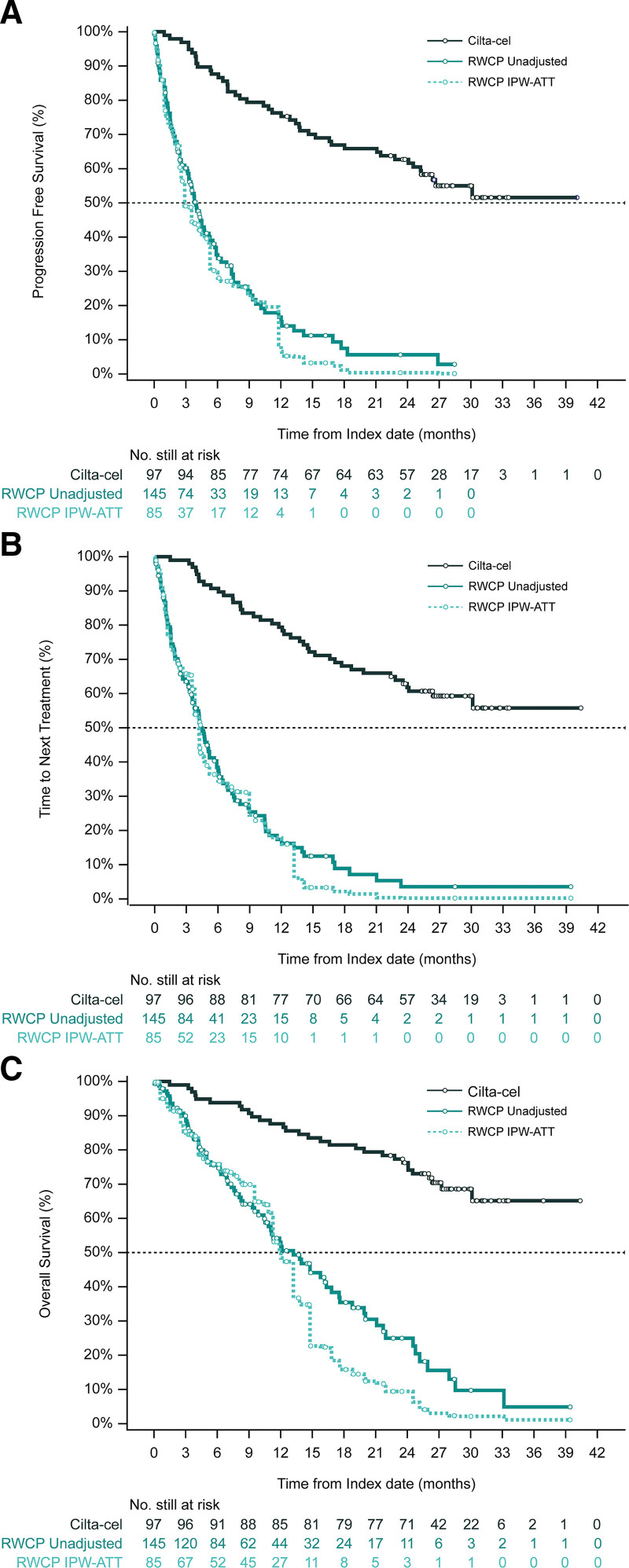
**Unadjusted and IPW-ATT adjusted Kaplan-Meier curves, PFS (A), TTNT (B), and OS (C), infused/aligned populations.** Unweighted Kaplan-Meier curves in the cilta-cel and RWCP groups for the infused/aligned population are shown for (A) PFS; (B) TTNT; and (C) OS. IPW-ATT adjusted Kaplan-Meier curves for the reweighted RWCP group are also presented. For all endpoints, separation between curves indicative of superior benefits with cilta-cel is apparent. ATT = average treatment effect in the treated population; CI = confidence interval; HR = hazard ratio; IPW = inverse probability weighting; OS = overall survival; PFS = progression-free survival; RWCP = real-world clinical practice; TTNT = time to next treatment.

Median TTNT for cilta-cel was not yet reached, while the observed and ATT-reweighted median TTNT for the RWCP cohort were 4.4 and 4.2 months, respectively. Figure 2B presents the unadjusted and adjusted Kaplan-Meier curves for TTNT for both intervention groups; the adjusted HR for cilta-cel versus RWCP was 0.11 [0.07-0.17].

Median OS was not yet reached in the cilta-cel group, while the observed and ATT-reweighted median OS for RWCP were 13.2 and 12.0 months, respectively. Figure 2C presents Kaplan-Meier curves for OS in both groups. The adjusted HR was, similar to other endpoints, significantly in favor of cilta-cel (HR = 0.13 [0.08-0.21]; *P* < 0.0001). Comparative analyses of PFS, TTNT, and OS within the enrolled population showed similar findings.

Sensitivity analyses performed for all 5 outcomes using multivariable regression, and also by adjusting for an expanded set of covariates, produced analogous clinical interpretations regarding the effectiveness of cilta-cel (see Suppl. Section 7).

While therapies for patients with MM have improved in recent years, the unmet medical need for novel treatments remains high for TCE RRMM patients. CARTITUDE-1 demonstrated unprecedented response and survival of TCE patients treated with cilta-cel.^7^ To establish comparative evidence of cilta-cel’s benefits relative to other therapies, an external control group based on real-world data from the BELCOMM cohort was used. The comparative analyses were implemented using rigorous statistical methods to adjust for imbalances between both patient cohorts and related potential confounding bias. These results provide compelling evidence regarding the benefits of cilta-cel for the treatment of TCE RRMM patients and align with other recent analyses involving externally derived controls for CARTITUDE-1 from different countries, despite variability in the RWCP regimens.^9–14^ The current study demonstrated important gains with cilta-cel compared to RWCP for ORR, ≥VGPR, PFS, TTNT, and OS. Patients treated with cilta-cel are 3.0 and 11.5 times more likely to reach ORR and ≥VGPR, respectively, and had substantial improvements in PFS (by 88%), TTNT (by 89%), and OS (by 87%).

In summary, the adjusted comparisons presented here reflect clinically important and statistically significant improvement in outcomes from cilta-cel in patients with TCE RRMM compared to RWCP based on representative data from Belgium and illustrate cilta-cel’s potential to address the high unmet therapeutic needs of this heavily pretreated population.

## AUTHOR CONTRIBUTIONS

Authors contributed to the manuscript as follows: Conceptualization: MD, MCV, SV, SVH, EM, JD, BH, WD, SA. Methodology: SVH, JD, FG, SV, DS, BH. Software: JD, FG, DS. Formal analysis: JD, FG. Investigation: MD, MCV, JD, NM, AV, IV, JD, FG, SV. Data curation: JD, FG. Writing—original draft preparation: MD, MCV, SV, EM, FG, JD, BH, SA. Writing—review and editing: MD, MCV, JD, NM, AV, IV, SV, SVH, EM, DS, JD, FG, BH, SD, WD, SA. Visualization: FG, JD. Supervision: MCV, MD, SA, SV, EM, SD, FG, JD, BH. Project administration: SV, EM, SVH. All authors have read and agreed to the published version of the manuscript.

## DISCLOSURES

The authors declare the following conflicts of interest: M-CV has served on advisory committees for Amgen, Takeda, BMS-Celgene, Janssen Pharmaceutica, and Sanofi, and has received research funding from BMS-Celgene and Janssen Pharmaceutica. MD has received honoraria and research funding from Amgen, Celgene, Janssen, and Sanofi. JDe has served in a consulting role for Celgene, Janssen, Novartis, and Takeda. NM has served on advisory committees for Amgen, Takeda, BMS-Celgene, Janssen Pharmaceutica, and Sanofi. DS has served in a consulting role for Realidad bvba. SA has served on advisory committees for AbbVie, Astellas, AstraZeneca, BMS/Celgene, Janssen, Pfizer and has received research funding from AbbVie, Amgen BMS/Celgene, Janssen, Roche, Takeda. SV, SVH, EM, JDi, FG, BH, WD, and SD are employees of Janssen. AVdV and IVB have no relationships to disclose.

## SOURCES OF FUNDING

This study was funded by Janssen-Cilag NV, LLC and Legend Biotech USA Inc.

## Supplementary Material



## References

[1] Raab MS Cavo M Delforge M. Multiple myeloma: practice patterns across Europe. Br J Haematol. 2016;175:66–76. 27291397 10.1111/bjh.14193

[2] Gandhi UH Cornell RF Lakshman A. Outcomes of patients with multiple myeloma refractory to CD38-targeted monoclonal antibody therapy. Leukemia. 2019;33:2266–2275. 30858549 10.1038/s41375-019-0435-7 PMC6820050

[3] Dimopoulos MA Moreau P Terpos E. Multiple myeloma: EHA-ESMO Clinical Practice Guidelines for diagnosis, treatment and follow-up†. Ann Oncol Off J Eur Soc Med Oncol. 2021;32:309–322. 10.1016/j.annonc.2020.11.014 33549387

[4] Haefliger B Diels J Ghilotti F. Baseline characteristics and survival outcomes of patients with tri-exposed multiple myeloma in a German registry. Proceedings of the 2021 European Hematology Association Conference; Virtual Meeting; June 9–17 2021.

[5] Mehra M Vogel M Valluri S. Patient characteristics and treatment patterns in relapsed/refractory multiple myeloma patients after exposure to a proteasome inhibitor, an immunomodulatory agent and daratumumab. J Clin Oncol. 2020;38:e20540–e20540.

[6] Madduri D Berdeja J Usmani S. CARTITUDE-1: Phase 1b/2 study of Ciltacabtagene Autoleucel, a B-cell maturation antigen–directed chimeric antigen receptor T cell therapy, in relapsed/refractory multiple myeloma. Blood. 2020;136(Supp 1):22.

[7] Berdeja JG Madduri D Usmani SZ. Ciltacabtagene autoleucel, a B-cell maturation antigen-directed chimeric antigen receptor T-cell therapy in patients with relapsed or refractory multiple myeloma (CARTITUDE-1): a phase 1b/2 open-label study. Lancet Lond Engl. 2021;398:314–324. 10.1016/S0140-6736(21)00933-8 34175021

[8] ClinicalTrials.gov (Web Page). A study of JNJ-68284528, a chimeric antigen receptor T cell (CAR-T) therapy directed against B-cell maturation antigen (BCMA) in participants with relapsed or refractory multiple myeloma (CARTITUDE-1). Published online March 2020. https://clinicaltrials.gov/ct2/show/NCT03548207

[9] Costa L Lin Y Martin T. Cilta-cel versus conventional treatment in patients with relapse/refractory multiple myeloma. J Clin Oncol. 2021;39:8030.

[10] Weisel K Martin T Krishnan A. Comparative efficacy of Ciltacabtagene Autoleucel in CARTITUDE-1 vs physician’s choice of therapy in the long-term follow-up of POLLUX, CASTOR, and EQUULEUS clinical trials for the treatment of patients with relapsed or refractory multiple myeloma. Clin Drug Investig. 2022;42:29–41. 10.1007/s40261-021-01100-y PMC8755696 34822128

[11] Martin T Krishnan A Yong K. Comparison of outcomes with ciltacabtagene autoleucel (cilta-cel) in CARTITUDE-1 versus real-world standard of care (RW SOC) for patients with triple-class exposed relapsed/refractory multiple myeloma. J Clin Oncol. 2021;39(Suppl): 8045.

[12] Martin T Krishnan A Yong K. Comparative effectiveness of ciltacabtagene autoleucel in CARTITUDE-1 versus physician’s choice of therapy in the Flatiron Health multiple myeloma cohort registry for the treatment of patients with relapsed or refractory multiple myeloma. EJHaem. 2022;3:97–108. 35846215 10.1002/jha2.312 PMC9175662

[13] Merz M Goldschmidt H Hari P. Adjusted comparison of outcomes between patients from CARTITUDE-1 versus multiple myeloma patients with prior exposure to PI, Imid and Anti-CD-38 from a German Registry. Cancers. 2021;13:5996. 34885106 10.3390/cancers13235996 PMC8656798

[14] Mateos MV Weisel KJ Martin T. Ciltacabtagene Autoleucel for triple-class exposed multiple myeloma: adjusted comparisons of CARTITUDE-1 patient outcomes versus therapies from real-world clinical practice from the LocoMMotion Prospective Study, presented at the 63rd American Society of Hematology (ASH) Annual Meeting & Exposition; December 11–14, 2021. Published online 2021.

