# Supplementary material for: Ciltacabtagene Autoleucel for Patients With Triple-class Exposed Multiple Myeloma: Adjusted Comparison of CARTITUDE-1 Patient Outcomes Versus Real-world Clinical Practice
Source: Hemasphere. 2022 Nov 29;6(12):e813. doi: 10.1097/HS9.0000000000000813 (PMC9722575; doi:10.1097/HS9.0000000000000813)
Supplement: Supplementary file 1 [file hs9-6-e813-s001.docx]

**SUPPLEMENT TO:**

Ciltacabtagene autoleucel for patients with triple-class exposed multiple myeloma: adjusted comparison of CARTITUDE-1 patient outcomes versus real-world clinical practice in Belgium. Delforge M, Vekemans MC, Depaus J, Meuleman N, Van de Velde A, VandeBroek I, Vandervennet S, Van Hoorenbeeck S, Moorkens E, Strens D, Diels J, Ghilotti F, Haefliger B, Dalhuisen D, Deraedt W, Anguille S.

- Section 1: Detailed Description of Study Methods
- Section 2: Formation of Study Populations
- Section 3: Treatment regimens, BELCOMM cohort
- Section 4: Medical Resource Use in the RWCP Group
- Section 5: Pre-/Post-IPW Balance, IPW-ATT Analyses for modified ITT Populations
- Section 6: Propensity Score Distributions
- Section 7: Findings from Sensitivity Analyses

A dedicated list of references for this Supplement is provided at the end of the document.

**Section 1: Detailed Description of Study Methods**

1. **Data Sources**

***Patients Treated with Ciltacabtagene Autoleucel in CARTITUDE-1***

CARTITUDE-1 (NCT03548207) is an open-label, single arm phase 1b/2 clinical trial studying the safety and efficacy of cilta-cel in adult patients with triple-class exposed RRMM ^1^. In this study, 113 patients from centers within the United States were enrolled between July 2018 and October 2019 and underwent apheresis, with the collected T-cells being used to produce cilta-cel. Overall 97 patients were administered cilta-cel, while 16 patients did not receive infusions due to early withdrawal (n=5), progressive disease (n=2) or death (n=9). The findings presented in this study are based upon data from CARTITUDE-1 as of January 2022, with a median patient follow-up of 27.7 months ^2^.

***Patients Receiving Treatments from Real World Clinical Practice in BELCOMM***

The BELCOMM cohort, representing therapies administered in clinical practice obtained through a non-interventional retrospective chart review, was used as the comparator data source. As data in BELCOMM were collected retrospectively, patients could have initiated more than one LOT after having met study eligibility criteria. The study involved the review of medical charts and a total of 112 patients (237 LOTs) were lastly included from a total of seven academic and non-academic Belgian centers, with data from the time period between March 2017 and May 2021. The median patient follow-up was 16.6 months. The medical records included diagnostic information, treatment details captured during routine clinical care, and prognostic information at the initiation of each treatment line. All data were gathered through patient records. An electronic case report form (eCRF) captured extensive information regarding each patient (see **sub-section G below**). For the current study, patients who met the main eligibility criteria of CARTITUDE-1 were selected, i.e., if they (1) had been exposed to a PI, an IMiD and an anti-CD38 antibody as part of previous therapy (either from different monotherapies or combination regimens); (2) had received at least 3 prior lines of MM treatment regimens (RRMM as defined by IMWG consensus criteria); (3) received a subsequent therapy after becoming triple class exposed; and (4) had an ECOG score < 2 (observations with missing ECOG (n=103) were included and combined with ECOG of 1, as outcomes for both subgroups were comparable). Patients with prior history of central nervous system involvement or signs of meningeal involvement were not eligible. Note: CARTITUDE-1 eligibility criteria allowed the inclusion of tri-exposed patients with <3 prior lines of therapy (when patients were double refractory to an ImiD and a PI). However, all enrolled patients in CARTITUDE-1 had previously received ≥3 prior lines of treatment, and the same criteria to include treatment lines was applied to BELCOMM. Additional CARTITUDE-1 eligibility criteria, (for example, absence of cardiac conditions) could not be applied to the BELCOMM cohort, as these data are outside the scope of the data source.

1. **Analysis Populations and Design**

Given the retrospective nature of the data, patients within BELCOMM were allowed to contribute multiple LOTs to data analyses. Systematically using either the first eligible or last eligible LOT from patients receiving RWCP within BELCOMM would introduce selection bias, as line of therapy is known to be associated with the outcomes of interest ^3^. To avoid this source of bias, all available LOTs were used in data analyses, as long as the study eligibility criteria were met at the start of each LOT ^4,5^; the unit of observation of the external cohort was thus the LOT within RWCP patients.

Within the CARTITUDE-1 study, a total of 113 patients were enrolled and underwent apheresis. As described above, sixteen patients discontinued from the study between apheresis and the time of infusion with cilta-cel. To inform analyses, data from the group of 97 patients that received cilta-cel infusions in CARTITUDE-1 (97 LOTs; referred to as the *infused population* from here on) were compared with the set of 90 patients from BELCOMM who were progression-free and alive 52 days after treatment initiation (145 LOTs; referred to as the *aligned population* from here on), which represents the average number of days passed between apheresis and infusion in CARTITUDE-1. Additionally, data analyses were also performed based upon the 113 patients enrolled in CARTITUDE-1 (113 LOTs) and the corresponding set of 112 patients who were enrolled in BELCOMM (237 LOTs) (referred to as the *enrolled populations* from here on).

In CARTITUDE-1, patients’ index date was defined as the date of apheresis for the enrolled population, while the date of infusion was selected for the population of infused patients. Within the BELCOMM cohort, the index date for the enrolled population was selected to be the date when inclusion criteria were met, while the date of treatment initiation plus 52 days was selected as the index date for analyses involving the aligned population.

1. **Identification of Index Dates**

In CARTITUDE-1, patients’ index date (T_0_) was defined as the date of apheresis for the enrolled population, while the date of infusion was selected for the population of infused patients. Within the BELCOMM cohort, T_0_ for the enrolled population was selected to be the date when inclusion criteria were met, while the date of treatment initiation plus 52 days was selected as T_0_ for analyses involving the aligned population.

Given the retrospective nature of the data, patients within BELCOMM were allowed to contribute multiple LOTs to data analyses. Systematically using either the first eligible or last eligible LOT from patients receiving RWCP within BELCOMM would introduce selection bias, as line of therapy is known to be associated with the outcomes of interest ^3^. To avoid this source of bias, all available LOTs were used in data analyses, as long as the study eligibility criteria were met at the start of each LOT ^4,5^; the unit of observation of the external cohort was thus the LOT within RWCP patients.

1. **Baseline characteristics for population alignment**

Bias from confounding related to imbalances in clinically important baseline characteristics can be of particular concern when comparing outcomes between non-randomized populations, thereby requiring the use of statistical adjustment. Within this study, prognostic baseline variables were identified by consultation of clinical experts as well as a literature review. Next, the availability of these variables within the IPD of both data sources was established. After completion of these two steps, the following factors, available in both CARTITUDE-1 and BELCOMM, were adjusted for in comparative analyses: refractory status, extramedullary disease, time to progression on last regimen, number of prior LOTs, years since MM diagnosis, average duration of prior LOTs, age, sex, lactate dehydrogenase (LDH), hemoglobin and albumin. Values were measured at index date if available.

Complete data were available for patients from CARTITUDE-1 for the variables included in main analyses, whereas for BELCOMM, variables with a proportion of missing values less than 25% (albumin 18%, hemoglobin 14%, EMD 7%, LDH 3%) were handled using multiple imputation with chained equations. Both univariate and multivariable regression analyses were conducted to assess the prognostic strength of the aforementioned variables, and imbalances between the intervention groups were subsequently inspected using standardized mean differences (SMD); values with magnitude >0.2 were thought to be indicative of potentially important differences between intervention groups^6^. Three additional covariates of interest (MM type, ECOG performance status and cytogenetic risk) were added in sensitivity analyses in models for propensity scores and regression, however these were not considered in the base case scenario: cytogenetic risk was associated with a high degree of missingness in the BELCOMM cohort (43.0%), while the addition of MM type and ECOG in both modeling approaches worsened the degree of balance between populations.

1. **Outcome Measures**

Outcome measures were aligned between CARTITUDE-1 and BELCOMM. Evaluation of response outcomes as assessed by overseeing physicians in both CARTITUDE-1 and BELCOMM was used in data analyses. Two response measures, i.e., overall response rate (ORR) and very good partial response or better (≥VGPR), and three time-to-event endpoints, i.e., progression-free survival (PFS), time to next treatment (TTNT) and overall survival (OS), were compared between cilta-cel and RWCP. Response measures were defined according to the IMWG criteria ^7^. Complete response was not included as an outcome measure; this endpoint requires bone marrow sampling ^7^, which is not routinely assessed by Belgian hematologists for the patient population within the BELCOMM cohort according to the EHA-ESMO guidelines ^8^.

PFS for the CARTITUDE-1 population was defined as the time from index date to the date of progression (as defined per IMWG criteria) or death as assessed by study investigators, whichever occurred first. Patients not progressing and alive at time of data cut were censored at the last disease evaluation before the start of any subsequent antimyeloma therapy or retreatment with cilta-cel. PFS for the BELCOMM cohort was defined as the time from the index date to the date of progression, or death, whichever occurred first. For subjects who were alive and had not reported disease progression after the index date, data were censored at the start of subsequent line, if any, or at their last follow-up date.

TTNT was defined in both data sources as the time from the index date to the initiation of the next therapy line or death, whichever occurred first. Patients who were still alive and did not initiate a next therapy line at the time of data-cut were censored at the last date upon which they were known to be alive.

OS for both data sources was defined as the time from the index date to the date of the subject’s death. Patients that were alive or whose vital status was unknown at the time of datacut were censored at the date they were last known to be alive.

1. **Statistical Methods**

Comparative analyses between cilta-cel and RWCP were performed using individual patient data (IPD) from the CARTITUDE-1 and BELCOMM cohorts. Adjusted comparisons for all outcomes were performed for both the infused/aligned and enrolled populations, and results from both unadjusted and adjusted analyses are presented.

Differences in the distributions of baseline variables between groups were adjusted through inverse probability weighting (IPW) analyses. In a first step, propensity score based inverse probability weights (IPW), estimated with a multivariable logistic regression including all available baseline characteristics based on pooled data for both cohorts, were generated for the BELCOMM patients, such that the weighted BELCOMM population was well balanced with CARTITUDE-1 on the baseline characteristics. Specifically, RWCP patients were assigned weights of *p/(1-p)*, where *p* is the propensity score representing the probability for patients to belong to the CARTITUDE-1 cohort conditional on their baseline characteristics.

Patients with multiple treatment lines initiated after fulfilling the inclusion criteria contributed multiple times to the analyses. Correlation of observations within patients was corrected using robust standard errors. Comparative effectiveness between cilta-cel and RWCP was evaluated using weighted logistic regression for binary outcomes. Odds ratios (OR) and response-rate ratios (RR) (with corresponding 95% confidence intervals (CI)) were estimated from the same statistical model ^9^. For time-to-event outcomes, weighted Cox proportional hazards (PH) models were used to estimate hazard ratios (HR) with corresponding 95% CIs. As an additional sensitivity analysis, comparative effectiveness for response and time to event endpoints were estimated based on multivariable logistic regression and multivariable Cox PH regression analyses including the same baseline characteristics.

To assess the validity of the Cox proportional hazards model used to analyse time-to-event endpoints, the assumption of proportional hazards was assessed by visual inspection of the log-cumulative hazard plot and the Schoenfeld residuals plot, as well as by performance of the Grambsch-Therneau test ^10^ (a p-value <0.05 was considered to determine the absence of proportionality). Visual assessments were also performed to evaluate the shape of the curves over time. All data analyses in the current study were carried out using SAS 9.4 (SAS Institute, Cary, North Carolina) and R version 4.0.3 (R Foundation for Statistical Computing, Vienna, Austria).

1. **Summary of Characteristics Collected for the BELCOMM Population**

- **Patient / disease characteristics at diagnosis:** Year of birth; Gender; Date of diagnosis; Type of myeloma isotype (Ig G/A/M/D-K/l); SD stage ISS stage I/II/III-A/B at diagnosis; Cytogenetics: FISH; del17p; t(4;14); t(14;16); t(14;:20); 1p/1q; Karyotype; Prior autologous stem cell transplant (Yes/no); Date from diagnosis to tri-exposure/ CARTITUDE-1 eligible date; Immunoglobulin sub type (light chain and M-protein type); Treatments prior to the start of the observation period (specify triple-, quad-, penta-exposed); Number of prior LOTs; Cytogenetic risk factors at diagnosis; Lactate dehydrogenase levels at diagnosis; Comorbidities and Comorbidity score at diagnosis; ECOG status at diagnosis; Osteolytic lesions (yes/no) at diagnosis; Myeloma associated osteopenia (yes/no) at diagnosis; Extramedullary plasmacytomas (yes/no) at diagnosis
- **At start of observation period:** Weight /Height; ECOG; Lactate dehydrogenase levels, Albumin, hemoglobin, Estimated Glomerular Filtration Rate (eGFR), creatinine, potassium, calcium, urea nitrogen; Comorbidities and Comorbidity score; Extramedullary plasmacytomas ≥1: yes, no; High-risk cytogenetic profile; Previous therapies for MM; Best response to previous treatments; Treatment duration of current line; Refractory status defined as patients who are refractory to a PI+IMiD, PI+CD38, or IMiD+CD38. Double-, triple-, quad-, and penta-refractory status at the start of the observation period.
- **During observation:** All following characteristics including: Weight; Start date; End date; Reason for discontinuation; Hospitalizations; Reasons for hospitalization; Date of admission; Date of discharge; Last contact date; Best response; Date of best response; Survival; If deceased - Date of death and Reason.
- **Medical resource utilization:** Medical resource utilization was collected during the treatment period and include number of hematologist visits, tests, day clinic visits, procedures (transfusion), hospitalizations and length of stay.

**Section 2: Formation of Study Populations**


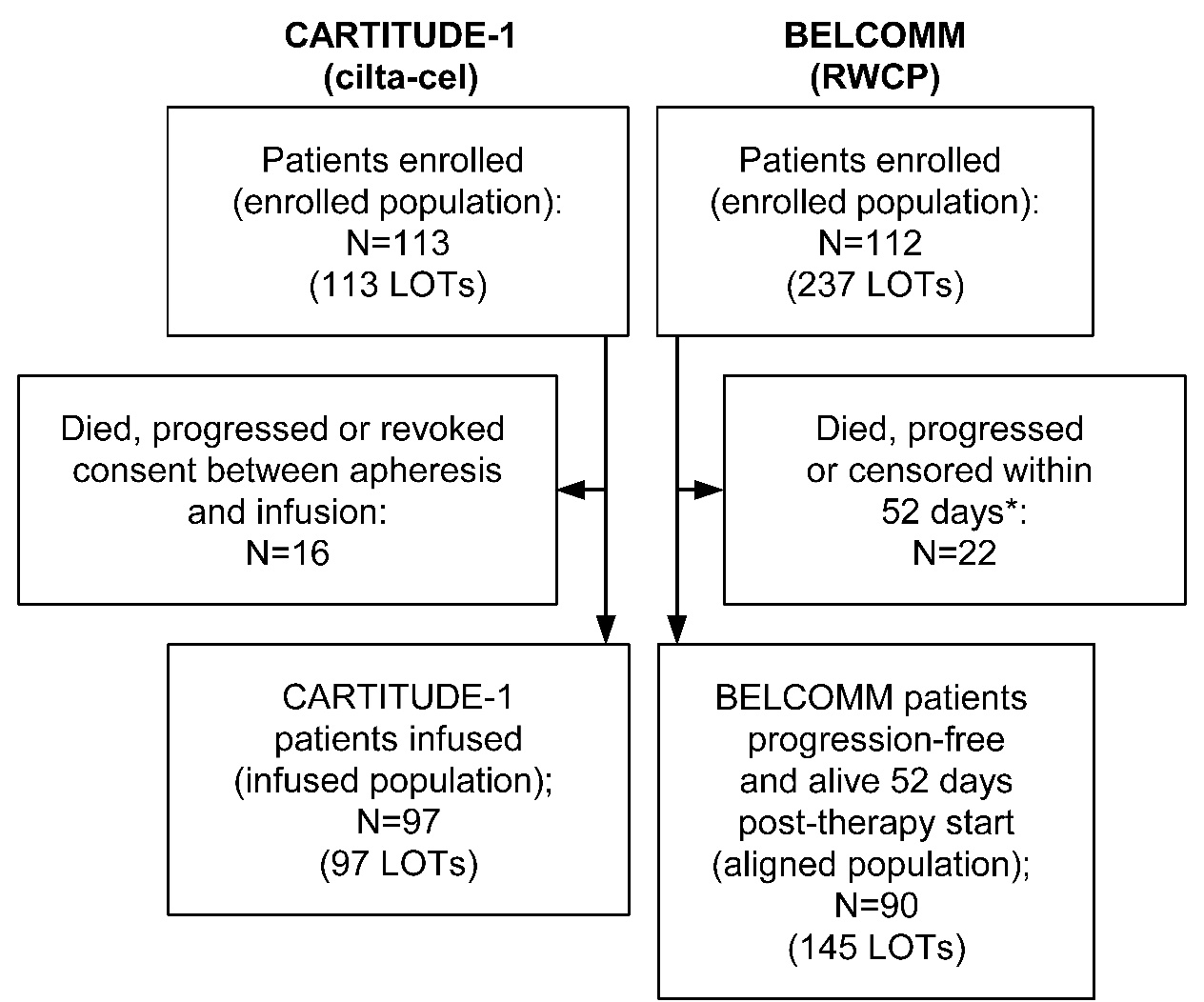


*****52 days was the mean duration between apheresis and infusion of cilta-cel within CARTITUDE-1.

**Abbreviations:** LOT, line of therapy.

**Section 3: Treatment Regimens, BELCOMM cohort**

| **Treatment Regimen** | **% of LOTs, Aligned Population** | **% of LOTs,**  **Enrolled Population** |
| --- | --- | --- |
| Carfilzomib, dexamethasone (Kd) | 14.5% | 13.9% |
| Pomalidomide, cyclophosphamide, dexamethasone/prednisone (PCd) | 10.3% | 10.5% |
| Pomalidomide, bortezomib, dexamethasone (PVd) | 5.5% | 5.5% |
| Ixazomib, lenalidomide, dexamethasone (IRd) | 8.3% | 5.5% |
| Elotuzumab, lenalidomide, dexamethasone (ERd) | 3.5% | 5.1% |
| Daratumumab, lenalidomide, dexamethasone (DRd) | 5.5% | 4.6% |
| Pomalidomide, dexamethasone (Pd) | 5.5% | 5.1% |
| Carfilzomib, lenalidomide, dexamethasone (KRd) | 4.1% | 3.8% |
| Daratumumab, cyclophosphamide, dexamethasone (DCd) | 2.1% | 3.4% |
| Bendamustine, prednisone | 2.1% | 3.4% |
| Cyclophosphamide, dexamethasone (Cd) | 3.5% | 2.5% |
| Bortezomib, dexamethasone + chemotherapy (Vd + chemo) | 0.7% | 1.7% |
| Bortezomib, dexamethasone (Vd) | 2.1% | 1.7% |
| Clinical trial/medical need program/compassionate use program    Belantamab mafodotin - alone or in combination (ant-BCMA)  Teclistamab (anti-BCMA)  Talquetamab  Selinexor- alone or in combination  Venetoclax - alone or in combination  Iberdomide  Melflufen | 5.0%  0%  0%  1.4%  0.7%  0%  0% | 5.0%  0.4%  0.4%  1.3%  0.8%  0.4%  0.4% |
| Other regimens (n = 41 unique regimens)  Daratumumab, cyclophosphamide, dexamethasone  Cyclophosphamide, dexamethasone  Bortezomib, dexamethasone, chemotherapy  Bortezomib, dexamethasone  Prednisone, melfalan  Daratumumab  Bortezomib, dexamethasone, venetoclax  Chemotherapy  DL-PACE  Daratumumab, cyclophosphamide  Carfilzomib, cyclophosphamide, dexamethasone  Carfilzomib, pomalidomide, dexamethasone  Daratumumab, bortezomib, dexamethasone  Daratumumab, pomalidomide, dexamethasone  Ixazomib  Ixazomib, cyclophosphamide, dexamethasone  Ixazomib, dexamethasone  Lenalidomide, dexamethasone  Pomalidomide  Bortezomib, doxorubine  Ixazomib, Lenalidomide  Ixazomib, pomalidomide, medrol  Pom+cyclo+dexa (PCd)/Pom+cyclo+prednisone  Second autologue transplantation  V(T)D-PACE  Venetoclax, daratumumab  Bortezomib, lenalidomide, dexamethasone  Carfilzomib, lenalidomide, dexamethasone, cyclophosphamide  Daratumumab, Melfalan, dexamethasone  Daratumumab, bortezomib, cyclophosphamide  Daratumumab, ixazomib  Daratumumab, ixazomib, pomalidomide  Daratumumab, lenalidomide, cyclophosphamide  Elotuzumab  Elotuzumab, ixazomib  Ixazomib, lenalidomide, cyclophosphamide, dexamethasone  Ixazomib, pomalidomide, cyclophosphamide, dexamethasone  Lenalidomide  Lenalidomide, elotuzumab  Panobinostat, bortezomib, dexamethasone  Pomalidomide, ixazomib, dexamethasone  Venetoclax, daratumumab, chemotherapy | 2.1%  0%  1.4%  0%  0.7%  1.4%  0%  1.4%  0.7%  0.7%  0%  0%  0.7%  0.7%  1.4%  1.4%  1.4%  0.7%  0.7%  0%  0.7%  0%  0.7%  0.7%  0%  0%  0%  0%  0%  0.7%  0.7%  0%  0.7%  0%  0.7%  0.7%  0.7%  0.7%  0%  0.7%  0%  0% | 3.4%  2.5%  1.7%  1.7%  1.7%  1.3%  1.3%  0.8%  0.8%  0.8%  0.8%  0.8%  0.8%  0.8%  0.8%  0.8%  0.8%  0.8%  0.8%  0.4%  0.4%  0.4%  0.4%  0.4%  0.4%  0.4%  0.4%  0.4%  0.4%  0.4%  0.4%  0.4%  0.4%  0.4%  0.4%  0.4%  0.4%  0.4%  0.4%  0.4%  0.4%  0.4% |

**Prior Exposure to Immunomodulatory Drugs, RWCP Group**

| **Previous Immunomodulatory Drugs** | **Aligned Population**  **(N=145 LOTs)** | **Enrolled Population**  **(N=237 LOTs)** |
| --- | --- | --- |
| *Lenalidomide*  Exposed  Refractory | 142 (97.9%)  118 (81.4%) | 255 (94.9%)  189 (79.8%) |
| *Daratumumab*  Exposed  Refractory | 145 (100.0%)  129 (89.0%) | 237 (100.0%)  216 (91.1%) |
| *Bortezomib*  Exposed  Refractory | 144 (99.3%)  78 (53.8%) | 236 (99.6%)  131 (55.3%) |
| *Lenalidomide, daratumumab and bortezomib*  Exposed  Refractory | 141 (97.2%)  60 (41.4%) | 224 (94.5%)  101 (42.6%) |

Data for cilta-cel regarding both prior exposures and refractoriness have been reported previously (Berdeja et al, 2021).

**Section 4: Medical Resource Use in the RWCP Group**

| **Reason for Hospitalization** | **Aligned Population**  **(N=151 hospitalizations in 90 patients)** | **Enrolled Population**  **(N=178 hospitalizations in 112 patients)** |
| --- | --- | --- |
| Infection | 55 (36.4%) | 63 (35.4%) |
| Progressive disease | 47 (31.1%) | 53 (29.8%) |
| Pain | 27 (17.9%) | 30 (16.8%) |
| Adverse event | 26 (17.2%) | 32 (18.0%) |
| Treatment Administration | 24 (15.9%) | 32 (18.0%) |
| RBC Transfusion | 22 (14.6%) | 29 (16.3%) |
| Platelet Transfusion | 12 (8.0%) | 17 (9.6%) |
| Renal insufficiency | 9 (6.0%) | 11 (6.2%) |
| Fracture | 4 (2.7%) | 5 (2.8%) |
| Cytopenia | 2 (1.3%) | 3 (1.7%) |
| Other | 35 (23.2%) | 43 (24.2%) |

NOTE: hospitalizations could be associated with more than one reason for admission

**Number of Hospitalizations, Aligned Population:**

**Per patient (N=90):**

| **# hospitalizations** | **# and % of patients** | **Additional Information** |
| --- | --- | --- |
| 0 | 37 (41.1%) | - Total of 151 hospitalizations - # hospitalizations per patient:   - Mean 1.68; Median 1.00 (range 0-8) - Length of stay (N=146; 5 with missing discharge date)   - Mean 11.48 days; Median 8.00 (IQR 5.00 to 14.00) |
| 1 | 17 (18.9%) |  |
| 2 | 10 (11.1%) |  |
| 3 | 8 (8.9%) |  |
| 4 | 8 (8.9%) |  |
| 5 | 4 (4.4%) |  |
| 6 | 5 (5.6%) |  |
| 8 | 1 (1.1%) |  |

**Per line of therapy (N=145):**

| **# hospitalizations** | **# and % of patients** | **Additional Information** |
| --- | --- | --- |
| 0 | 89 (61.4%) | - Total of 101 hospitalizations - # hospitalizations per patient:   - Mean 0.70; Median 0.00 (range 0-8) |
| 1 | 20 (20.0%) |  |
| 2 | 19 (13.1%) |  |
| 3 | 4 (2.8%) |  |
| 4 | 2 (1.4%) |  |
| 6 | 1 (0.7%) |  |
| 8 | 1 (0.7%) |  |

**Number of Hospitalizations, Enrolled Population:**

**Per patient (N=112 patients):**

| **# hospitalizations** | **# and % of patients** | **Additional Information** |
| --- | --- | --- |
| 0 | 45 (40.2%) | - Total of 178 hospitalizations - # hospitalizations per patient:   - Mean 1.59; Median 1.00 (range 0-8) - Length of stay (N=171; 7 with missing discharge date)   - Mean 11.47 days; Median 8.00 (IQR 5.00 to 14.00) |
| 1 | 25 (22.3%) |  |
| 2 | 12 (10.7%) |  |
| 3 | 9 (8.0%) |  |
| 4 | 11 (9.8%) |  |
| 5 | 4 (3.6%) |  |
| 6 | 5 (4.5%) |  |
| 8 | 1 (0.9%) |  |

**Per line of therapy (N=237):**

| **# hospitalizations** | **# and % of patients** | **Additional Information** |
| --- | --- | --- |
| 0 | 143 (60.3%) | - Total of 156 hospitalizations - # hospitalizations per patient:   - Mean 0.66; Median 0.00 (range 0-8) |
| 1 | 56 (23.6%) |  |
| 2 | 26 (11.0%) |  |
| 3 | 7 (3.0%) |  |
| 4 | 2 (0.8%) |  |
| 5 | 1 (0.4%) |  |
| 6 | 1 (0.4%) |  |
| 8 | 1 (0.4%) |  |

**Section 5: Pre-/Post-IPW Balance, IPW-ATT Analyses for Infused/Aligned Populations**

Using Base Model (refractory status, extramedullary disease, time to progression on prior line, # prior lines, years since diagnosis, average duration of prior lines, age, sex, hemoglobin, LDH, albumin)

Group Demographic Balance Before and After IPW-ATT Weighting (Infused/Aligned Populations)

| **Variable** | **Categories** | **Before IPW** | | | **After IPW-ATT Weighting** | | |
| --- | --- | --- | --- | --- | --- | --- | --- |
|  |  | **CARTITUDE-1,**  **%**  (N=97) | **Belcomm Cohort,**  **%**  (N=145) | **SMD** | **CARTITUDE-1,**  **%**  (N=97) | **BELCOMM Cohort,**  **%**  (N=85) | **SMD** |
| **Refractory status** | Penta refractory  Quad refractory  Triple refractory  <= Double refractory | 42.3%  37.1%  8.2%  12.4% | 13.1%  24.8%  20.7%  41.4% | 1.00 | 42.3%  37.1%  8.2%  12.4% | 41.2%  37.6%  8.4%  12.8% | 0.05 |
| **EMD** | Yes  No | 13.4%  86.6% | 14.5%  85.5% | 0.03 | 13.4%  86.6% | 5.7%  94.3% | -0.26 |
| **Time to progress on last prior line (months)** | < 4  4+ | 49.5%  50.5% | 42.1%  57.9% | -0.15 | 49.5%  50.5% | 47.8%  52.2% | -0.03 |
| **Number of prior LOTs** | ≤ 4  5+ | 34.0%  66.0% | 43.0%  57.0% | 0.18 | 34.0%  66.0% | 25.4%  74.6% | -0.19 |
| **Years since MM diagnosis** | <6  6+ | 46.4%  53.6% | 47.6%  52.4% | 0.02 | 46.4%  53.6% | 30.8%  69.2% | -0.32 |
| **Average duration of prior lines (months)** | <8.14  8.14 to <11.76  11.76 to <17.61  >17.61 months | 20.6%  22.7%  27.8%  28.9% | 15.9%  19.3%  31.0%  33.8% | 0.19 | 20.6%  22.7%  27.8%  28.9% | 14.0%  24.2%  31.7%  30.1% | 0.19 |
| **Age (years)** | <65  65+ | 63.9%  36.1% | 31.7%  68.3% | -0.68 | 63.9%  36.1% | 54.4%  45.6% | -0.19 |
| **Sex** | Male  Female | 58.8%  41.2% | 49.0%  51.0% | -0.20 | 58.8%  41.2% | 52.7%  47.3% | -0.12 |
| **LDH** | <280 U/L  280+ U/L | 87.6%  12.4% | 84.1%  15.9% | -0.10 | 87.6%  12.4% | 88.3%  11.7% | 0.02 |
| **Hemoglobin** | <12 g/L  12+ g/L | 92.8%  7.2% | 78.6%  21.4% | -0.41 | 92.8%  7.2% | 95.9%  4.1% | 0.14 |
| **Albumin** | <3.5 g/dL  3.5+ g/dL | 55.7%  44.3% | 24.1%  75.9% | -0.68 | 55.7%  44.3% | 47.1%  52.9% | -0.17 |
| **Summary Diagnostics** | | | | | | | |
| **Mean SMD** | | 0.33 | | | 0.15 | | |
| **% SMDs > 0.2** | | 4 / 11 = 36.4% | | | 2 / 11 = 18.2% | | |

The pre-weighting and post-weighting distributions of demographics by intervention group are shown. SMDs >0.2 are considered to indicate differences between groups.

**Section 6: Propensity Score Distributions**

**Propensity Scores Before and After Weighting, Infused/Aligned populations (all LOTs)**

| 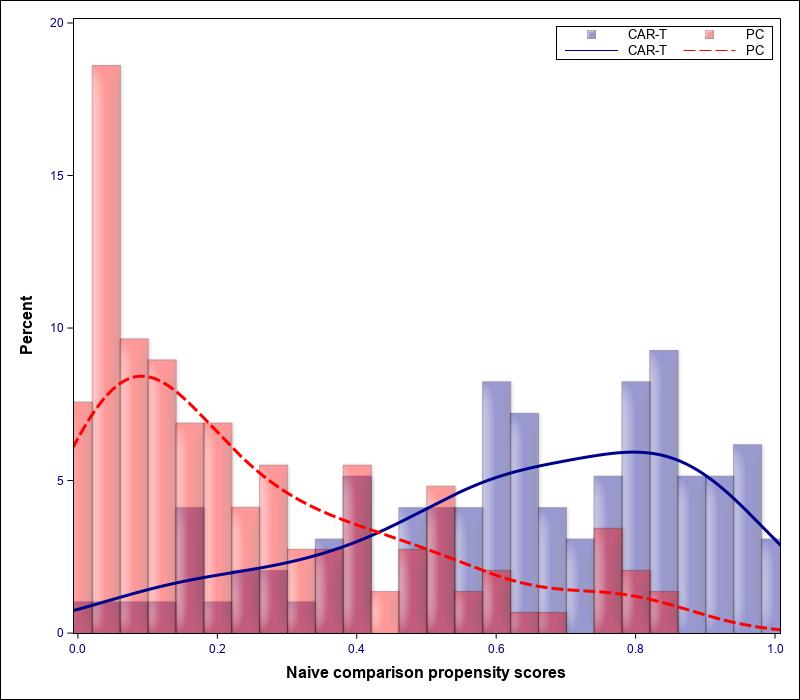 | **Naïve Comparison** |
| --- | --- |

| 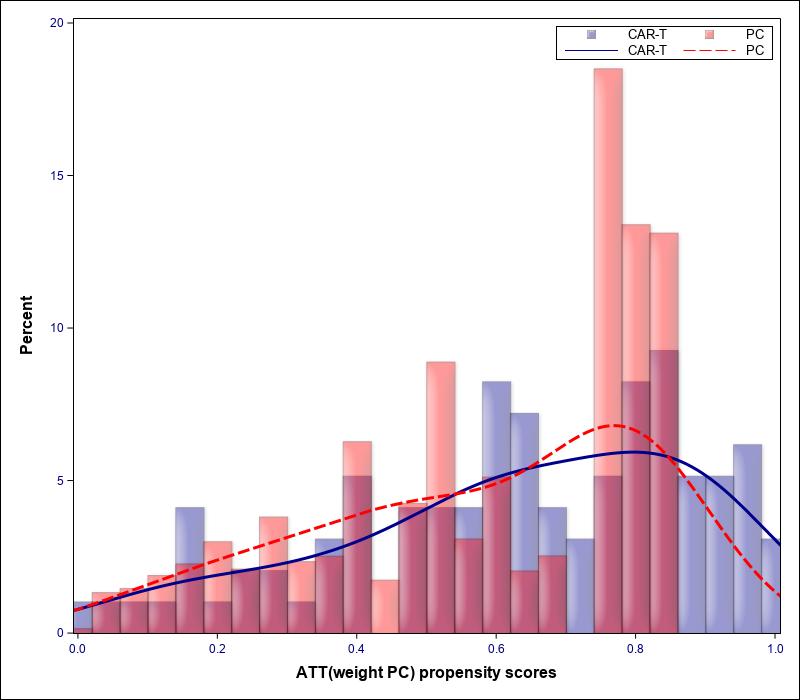 | **IPW-ATT** |
| --- | --- |

**Propensity Scores Before and After Weighting, Enrolled populations (all LOTs)**

| 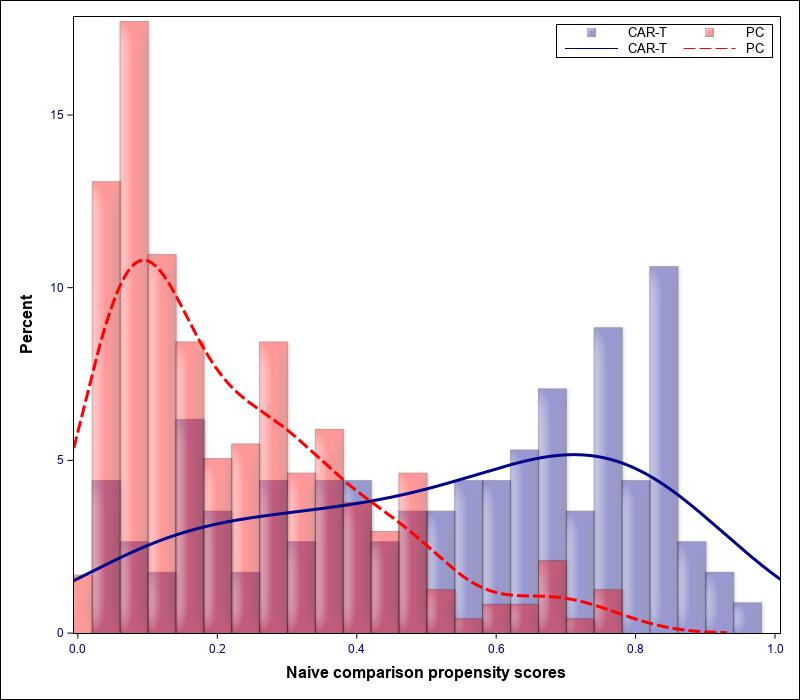 | **Naïve comparison** |
| --- | --- |

| 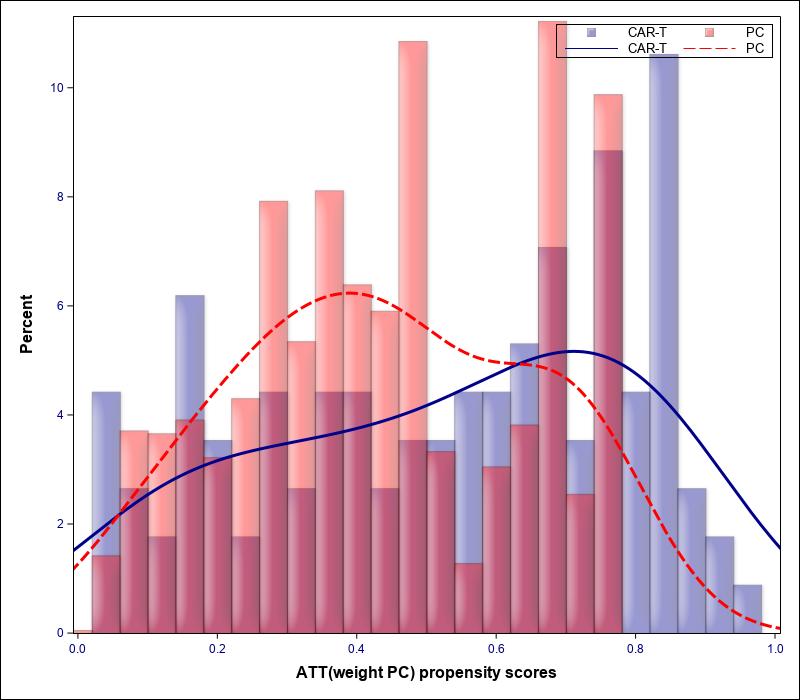 | **IPW-ATT** |
| --- | --- |

**Section 7: Findings from Sensitivity Analyses**

**Summary of Unadjusted and Adjusted Comparisons for Response (Panel A) and Time-to-Event (Panel B) Outcomes, Main Set of Covariates**


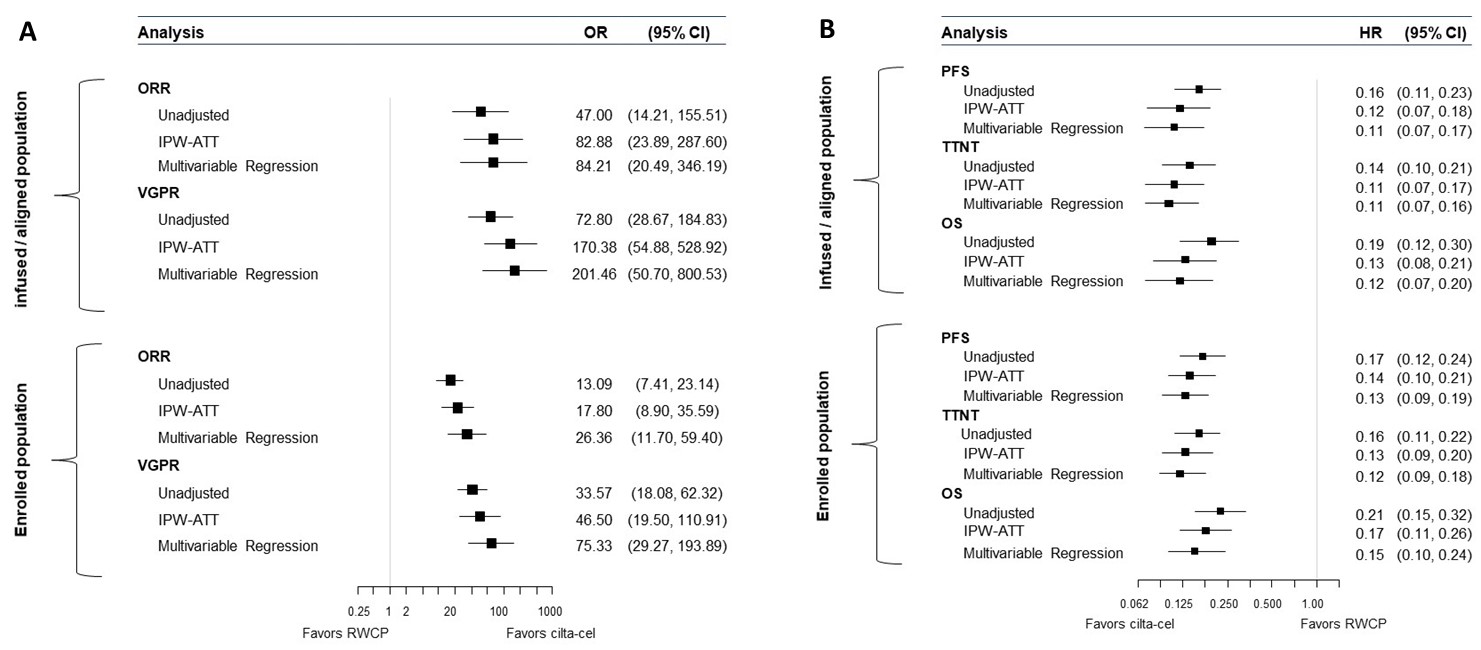


Forest plots present odds ratios and hazard ratios comparing cilta-cel and RWCP with corresponding 95% CIs for (A) ORR, ≥VGPR and (B) PFS, TTNT and OS for the infused/aligned and enrolled populations. Both the unadjusted comparison as well as adjusted comparisons derived using IPW methods and multivariable regression models are shown. Across methods, findings for all endpoints consistently suggest increased benefits with cilta-cel compared to RWCP.

**Abbreviations:** ATT, average treatment effect in the treated population; CI, confidence interval; HR, hazard ratio; IPW, inverse probability weighting; OR, odds ratio; ORR, overall response rate; PFS, progression-free survival; RWCP, real-world clinical practice; VGPR, very good partial response.

Findings from sensitivity analyses that were performed using an extended set of clinical covariates (adding cytogenetic risk, MM type and ECOG PS) are presented below for response measures and time-to-event outcomes.

**Table: Adjusted Comparisons of Clinical Response, Extended Variable Set**

| **Outcome** | **IPW-ATT** | | | **Multivariable logistic regression** | |
| --- | --- | --- | --- | --- | --- |
|  | **OR** | **RR** | **OR** | |  |
| **Infused / Aligned Population** | | | | |  |
| **ORR** | 250.06  (67.82, 921.97) | 8.34  (4.92, 14.18) | 91.77  (20.68, 407.24) | |  |
| **≥VGPR** | 456.44  (115.26, 1807.60) | 19.21  (9.82, 37.58) | 387.44  (72.83, 2061.21) | |  |
| **Enrolled Population** | | | | |  |
| **ORR** | 31.98  (15.18, 67.37) | 4.43  (2.70, 7.27) | 26.52  (10.92, 64.40) | |  |
| **≥VGPR** | 81.70  (29.96, 222.85) | 9.34  (5.06, 17.24) | 76.04  (26.85, 215.35) | |  |

**Abbreviations**: ATT, average treatment effect in the treated population; IPW, inverse propensity weighting; OR, odds ratio; ORR, overall response rate; RR, response ratio; VGPR, very good partial response rate.

**Table: Adjusted Comparisons of PFS, TTNT and OS (HR and 95% CI), Extended Variable Set**

| **Outcome** | **IPW-ATT** | **Multivariable Cox**  **PH regression** |
| --- | --- | --- |
| **Infused / Aligned Population** | | |
| **PFS** | 0.14  (0.07, 0.28) | 0.11  (0.07, 0.19) |
| **TTNT** | 0.13  (0.06, 0.26) | 0.11  (0.07, 0.18) |
| **OS** | 0.11  (0.06, 0.21) | 0.09  (0.05, 0.18) |
| **Enrolled Population** | | |
| **PFS** | 0.14  (0.09, 0.21) | 0.12  (0.08, 0.20) |
| **TTNT** | 0.14  (0.09, 0.21) | 0.12  (0.08, 0.19) |
| **OS** | 0.15  (0.09, 0.24) | 0.12  (0.07, 0.21) |

**Abbreviations**: ATT, average treatment effect in the treated population; HR, hazard ratio; IPW, inverse propensity weighting; OS, overall survival; PFS, progression-free survival; TTNT, time to next treatment.

**Overview of Group Demographic Balance Before and After IPW-ATT Weighting, Extended Model (Infused/Aligned Populations)**

| **Variable** | **Categories** | **Before IPW-ATT** | | | **After IPW-ATT** | | |
| --- | --- | --- | --- | --- | --- | --- | --- |
|  |  | **CARTITUDE-1,**  **%**  (N=97) | **Belcomm Cohort,**  **%**  (N=145) | **SMD** | **CARTITUDE-1,**  **%**  (N=97) | **BELCOMM Cohort,**  **%**  (N=102) | **SMD** |
| **Refractory status** | Penta refractory  Quad refractory  Triple refractory  <= Double refractory | 42.3%  37.1%  8.2%  12.4% | 13.1%  24.8%  20.7%  41.4% | 1.00 | 42.3%  37.1%  8.2%  12.4% | 51.8%  27.9%  12.6%  7.8% | 0.28 |
| **EMD** | Yes  No | 13.4%  86.6% | 14.5%  85.5% | 0.03 | 13.4%  86.6% | 2.1%  97.9% | -0.44 |
| **Time to progress on last prior line (months)** | < 4  4+ | 49.5%  50.5% | 42.1%  57.9% | -0.15 | 49.5%  50.5% | 30.2%  69.8% | -0.40 |
| **Number of prior LOTs** | ≤ 4  5+ | 34.0%  66.0% | 43.0%  57.0% | 0.18 | 34.0%  66.0% | 27.7%  72.3% | -0.14 |
| **Years since MM diagnosis** | <6  6+ | 46.4%  53.6% | 47.6%  52.4% | 0.02 | 46.4%  53.6% | 29.7%  70.3% | -0.34 |
| **Average duration of prior lines (months)** | <8.14  8.14 to <11.76  11.76 to <17.61  >17.61 months | 20.6%  22.7%  27.8%  28.9% | 15.9%  19.3%  31.0%  33.8% | 0.19 | 20.6%  22.7%  27.8%  28.9% | 17.9%  9.1%  19.2%  53.7% | 0.60 |
| **Age (years)** | <65  65+ | 63.9%  36.1% | 31.7%  68.3% | -0.68 | 63.9%  36.1% | 66.4%  33.6% | 0.05 |
| **Sex** | Male  Female | 58.8%  41.2% | 49.0%  51.0% | -0.20 | 58.8%  41.2% | 23.3%  76.7% | -0.77 |
| **LDH** | <280 U/L  280+ U/L | 87.6%  12.4% | 84.1%  15.9% | -0.10 | 87.6%  12.4% | 87.7%  12.3% | 0.001 |
| **Hemoglobin** | <12 g/L  12+ g/L | 92.8%  7.2% | 78.6%  21.4% | -0.41 | 92.8%  7.2% | 98.3%  1.7% | 0.27 |
| **Albumin** | <3.5 g/dL  3.5+ g/dL | 55.7%  44.3% | 24.1%  75.9% | -0.68 | 55.7%  44.3% | 32.3%  67.7% | -0.48 |
| **MM type** | IgA  IgG  Light chain  Other | 8.2%  58.8%  24.7%  8.2% | 20.0%  48.3%  28.3%  3.4% | 0.40 | 8.2%  58.8%  24.7%  8.2% | 4.1%  66.7%  18.8%  10.3% | 0.24 |
| **ECOG PS** | 0  1 | 40.2%  59.8% | 12.4%  87.6% | -0.67 | 40.2%  59.8% | 56.4%  43.6% | 0.33 |
| **Cytogenetic risk** | High  Low  Missing | 70.1%  23.7%  6.2% | 51.7%  6.2%  42.1% | 1.01 | 70.1%  23.7%  6.2% | 81.2%  11.4%  7.5% | 0.35 |
| **Summary Diagnostics** | | | | | | | |
| **Mean SMD** | | 0.41 | | | 0.34 | | |
| **% SMDs > 0.2** | | 7 / 14 = 50% | | | 11 / 14 = 78.6% | | |

The pre-weighting and post-weighting distributions of demographics by intervention group are shown. SMDs >0.2 are considered to indicate differences between groups.

**Abbreviations**: ATT, average treatment effect in the treated population; ECOG PS, Easter Co-operative Oncology Group Performance Status; EMD, extramedullary disease; LDH, lactate dehydrogenase; LOTs, lines of therapy; MM, multiple myeloma; SMD, standardized mean difference

**Reference List for Supplement**

1. Berdeja JG, Madduri D, Usmani SZ, et al. Ciltacabtagene autoleucel, a B-cell maturation antigen-directed chimeric antigen receptor T-cell therapy in patients with relapsed or refractory multiple myeloma (CARTITUDE-1): a phase 1b/2 open-label study. *Lancet Lond Engl*. 2021;398(10297):314-324. doi:10.1016/S0140-6736(21)00933-8

2. Martin T, Usmani S, Berdeja J, Jakubowiak A, Agha M, Cohen A et al. Updated Results From CARTITUDE-1: Phase 1b/2 Study of Ciltacabtagene Autoleucel, a B-cell Maturation Antigen–Directed Chimeric Antigen Receptor T Cell Therapy, in Patients With Relapsed/Refractory Multiple Myeloma. 9. In: ; 2021.

3. Backenroth D. How to choose a time zero for patients in external control arms. *Pharm Stat*. 2021;20(4):783-792. doi:10.1002/pst.2107

4. van der Laan M, Petersen M, Joffe M. History-adjusted marginal structural models and statically-optimal dynamic treatment regimens. *Int J Biostat*. 2005;1(1).

5. Petersen ML, Deeks SG, Martin JN, van der Laan MJ. History-adjusted marginal structural models for estimating time-varying effect modification. *Am J Epidemiol*. 2007;166(9):985-993. doi:10.1093/aje/kwm232

6. Yang D, Dalton J. A unified approach to measuring the effect size between two groups using SAS®. *Sas Glob Forum 2012*. Published online 2012.

7. Kumar S, Paiva B, Anderson KC, et al. International Myeloma Working Group consensus criteria for response and minimal residual disease assessment in multiple myeloma. *Lancet Oncol*. 2016;17(8):e328-e346. doi:10.1016/S1470-2045(16)30206-6

8. Dimopoulos MA, Moreau P, Terpos E, et al. Multiple myeloma: EHA-ESMO Clinical Practice Guidelines for diagnosis, treatment and follow-up†. *Ann Oncol Off J Eur Soc Med Oncol*. 2021;32(3):309-322. doi:10.1016/j.annonc.2020.11.014

9. Grant RL. Converting an odds ratio to a range of plausible relative risks for better communication of research findings. *BMJ*. 2014;348:f7450. doi:10.1136/bmj.f7450

10. Grambsch P, Therneau T. Proportional hazards tests and diagnostics based on weighted residuals. *Biometrika*. 1994;81(3):515-526.
